# EFCNet for small object detection in remote sensing images

**DOI:** 10.1038/s41598-025-09066-z

**Published:** 2025-07-01

**Authors:** Yutong Wang, Zhensong Li, Shiliang Zhu, Xiaotan Wei

**Affiliations:** Key Laboratory of the Ministry of Education for Optoelectronic Measurement Technology and Instrument, Beijing Information Science and Technology University, Beijing, 100192 China

**Keywords:** Engineering, Aerospace engineering

## Abstract

Object detection, as a crucial component of remote sensing image processing, has become one of the primary methods with the maturation of deep learning technologies. Nonetheless, detecting small objects in remote sensing images remains a significant challenge. Addressing this issue, this study proposes an enhanced network model based on You Only Look Once YOLOv5, aimed at improving the detection capabilities for small objects in remote sensing images. The model employs a novel backbone network, ODCSP-Darknet53, to enhance feature extraction efficiency, and incorporates the small object enhancement bi-directional feature pyramid network (STEBIFPN) structure in the neck region of the network for optimized scaling of small object information. Additionally, we have designed two distinct weighted fusion strategies to further boost the model’s performance in detecting small objects. In the detection head portion of the model, a four-head detection network specialized for small objects is constructed, and adaptively spatial feature fusion (ASFF) technology is introduced to optimize the recognition capabilities for small objects. Experiments conducted on the DOTA and DIOR datasets demonstrate that our model achieves an average precision mean of 75.9% and 80.5%, respectively, with the model’s parameters and computational requirements amounting to 13.4M and 30.2 GFLOPs, respectively. Compared to the original YOLOv5s model, our model exhibits significant performance improvements in detecting typical small objects such as Bridge and Ship. Thus, this research provides an effective solution for object detection in the field of remote sensing image processing.

**Supplementary Information:**

The online version contains supplementary material available at 10.1038/s41598-025-09066-z.

Remote sensing technology plays a significant role in both military and civilian domains, including traffic monitoring^1^, maritime rescue^2^, and aviation control^3^, making the analysis and processing of remote sensing images critically important. Compared to traditional images, remote sensing images are captured from high altitudes, which due to factors like angle and distance, results in more complex backgrounds and many small objects. The objects in remote sensing images often exhibit arbitrary orientations, large scale variations, highly uneven distributions, and large aspect ratios, all of which undeniably add to the challenges of object detection in these images^4–6^. Object detection, as a key aspect of remote sensing image processing, is extensively used in fields such as oceanography, urban planning, and agricultural cultivation. Current object detection methods include traditional machine learning algorithms, which are relatively cumbersome as they require manual selection and design of feature extraction algorithms to derive useful features from images for detection. Moreover, convolutional neural network (CNN) based methods have rapidly evolved and can be categorized into two-stage and one-stage detection methods. Two-stage detection methods first generate a set of candidate boxes, then classify and refine the positioning of these boxes. Common two-stage models include R-CNN^7^, Mask R-CNN^8^, and Faster R-CNN^9^. In contrast, one-stage detection methods skip the generation and filtering of candidate boxes, directly predicting the class and location of objects within the image. This approach, by simplifying the detection process, significantly speeds up processing and is suitable for real-time applications. Popular one-stage algorithms include the YOLO series^10–17^ and SSD^18^.

**Fig. 1:**
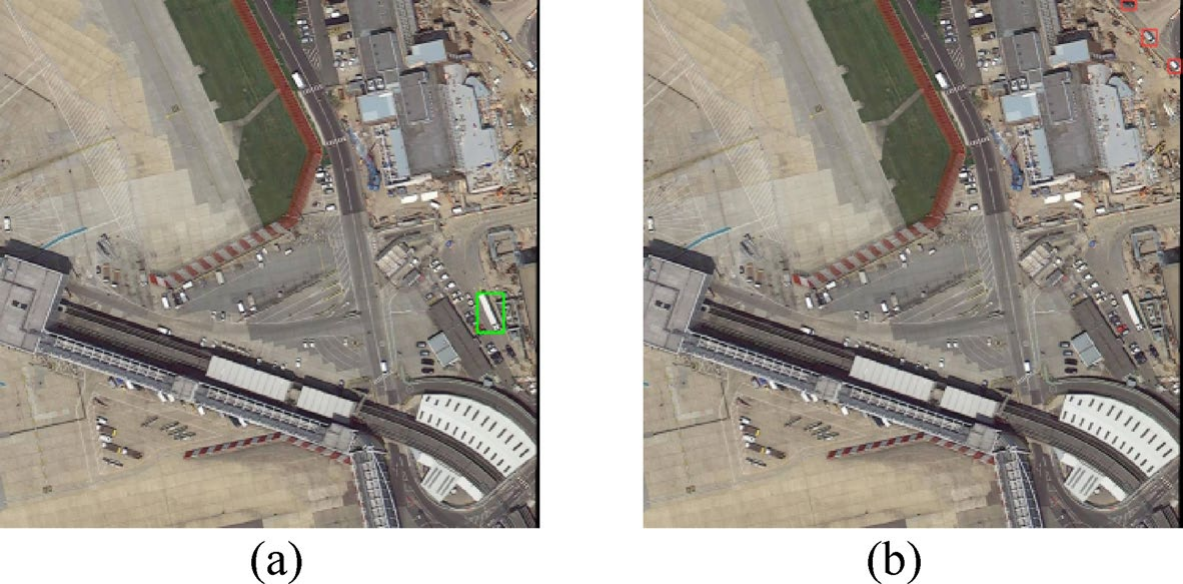
YOLOv5s detection results on DOTA dataset, Figure (**a**) shows the YOLO test results and figure (**b**) shows the real annotation box.

Among them, the YOLO series has been particularly effective in balancing accuracy with detection speed. However, there is still substantial room for improvement in the detection of complex remote sensing images. For instance, as shown in Figure. 1(a), the objects to be detected are small and densely packed, while Figure. 1(b) displays the detection results of YOLOv5, clearly indicating missed detections, reflecting the insufficiencies in extracting information from small objects.

In recent years, improvements to the YOLO series networks have achieved favourable results. Wu et al.^19^ eliminated unnecessary residual modules and introduced a refined residual coordinate attention module, replacing the average pooling operation, which enhanced the feature representation of densely packed small objects. They also used a differential evolution algorithm to generate anchor boxes of various scales, adapting to the diverse object sizes present in HRRSI. Xie et al.^20^ proposed a lightweight detection model named CSPPartial-YOLO. This model incorporates a partial hybrid dilated convolution (PHDC) module, combining hybrid dilated convolutions with partial convolutions to increase the receptive field at a lower computational cost. They also constructed the CSPPartialStage to enhance the detection capabilities for small, complexly distributed objects in remote sensing images. Yang et al.^21^ used the IMNet as the backbone feature extraction network, significantly enhancing feature extraction capabilities while reducing the parameter count. They employed the Slim-BiFPN for adaptive fusion of multi-scale features with fewer parameters. Liu et al.^22^ drew inspiration from residual networks to create YOLO-Extract, integrating Coordinate Attention into the network. They also combined hybrid dilated convolutions with a redesigned residual structure, enhancing the shallow layer feature and positional information extraction capabilities, and optimizing the feature extraction for objects of varying scales. Jiang et al.^23^ aimed to im-prove the detection accuracy for small objects by sutilizing the C3D module to fuse deep and shallow semantic information, optimizing the multi-scale issues of remote sensing objects, and introduced a feature extraction method based on the regin attention (RA) mechanism combined with the Swin Transformer backbone. Li et al.^24^ introduced the 3D attention mechanism SimAM, which adaptively weights each channel and three-dimensional spatial features, reducing interference from irrelevant information in complex scenarios and enhancing the detection of small objects. Cheng et al.^25^ proposed using 1D convolution in the efficient channel attention bottleneck module, which enhances the backbone’s feature extraction capability for small and elongated defects. Zhang et al.^26^ employed a combination of the Normalized Weighted Distance Loss small objects detection algorithm and the Wise Intersection Over Union loss function to replace the original loss function, thereby improving the small objects detection performance. Sharba, A et al.^27^ introduced an attention mechanism into YOLOv5, placed after the last layer of the backbone, and slightly increased the number of parameters to enhance the feature extraction capability of the backbone network. Guo et al.^28^ integrated SKAttention into the Backbone layer of the network to address the issue of overlapping and redundant information within the model. Yu et al.^29^ enhanced the model’s ability to fit targets by replacing the sigmoid linear unit (SiLU) activation function with the exponential linear unit (ELU). In addition, S.O. Slim et al.^30^ improved the detection accuracy of the YOLOv5 model by employing data augmentation and transfer learning techniques, Ma et al.^31^ proposed a Scale Decoupling Module (SDM) to enhance small object features by suppressing large object features in the shallow layers.

In the aforementioned methods, the network has been optimized for small object detection. However, due to the unique characteristics of remote sensing images, merely incorporating attention mechanisms and modifying the loss function to enhance model nonlinearity is insufficient to achieve satisfactory performance in remote sensing object detection. Therefore, the model proposed in this paper is specifically optimized for small remote sensing objects, with the following key improvements:

- In the backbone of the network, omni-dimensional dynamic convolution (ODConv)^32^ has been introduced at the shallow layers of the existing backbone network, forming the new ODCSP-Darknet53 backbone network. This improvement enhances the model’s capability to extract information from complex backgrounds and small objects in remote sensing images, facilitating deeper learning of small object features.
- In the neck region of the network, an efficient small object enhancement bi-directional feature pyramid network (STEBIFPN) structure is designed to optimize the fusion paths for small object information. This improved feature pyramid network structure facilitates more effective extraction and transmission of small object features, thereby enhancing the precision and efficiency of small object detection. Moreover, this structure employs two different weighted fusion methods, further optimizing the scaling of small object information while effectively extracting these details.
- To enhance the detection capabilities for extremely small objects, this study employs a four-head detection structure and specifically adds a detection head dedicated to extremely small objects. Simultaneously, the adaptively spatial feature fusion (ASFF)^33^ technology is introduced, which effectively integrates multi-scale information through detection heads of various sizes. This improves the model’s generalization ability and detection performance when handling objects of different sizes.

With the above improvements, the model proposed in this paper demonstrates significant performance improvement in small object detection in remote sensing images.

## Methodology

**Fig. 2:**
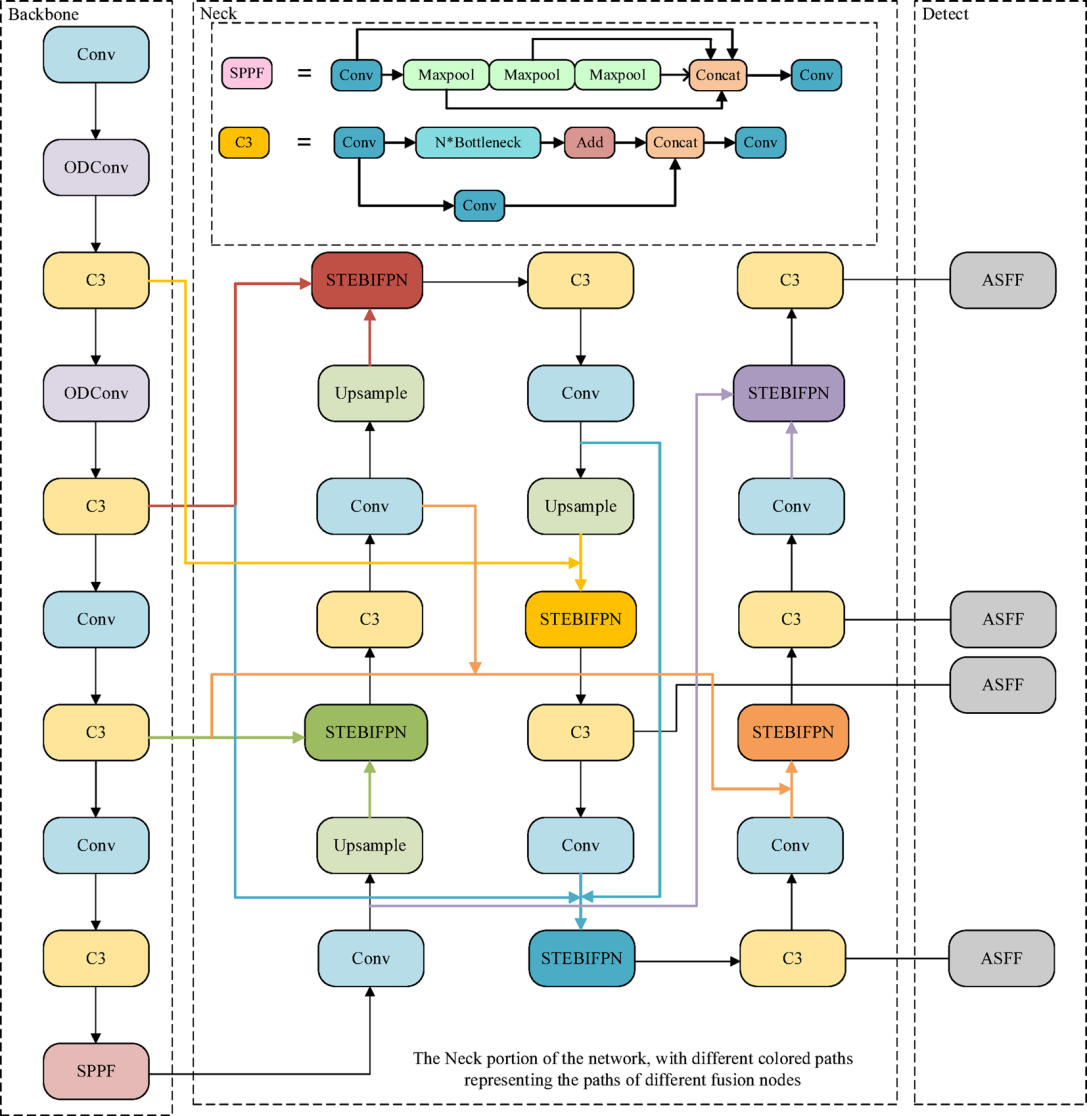
Network structure.

Figure. 2 illustrates the improved network model. In our enhanced feature convergence network (EFCNet), the backbone employs the ODCSP-Darknet53 architecture.

This structure optimizes the CSP-Darknet53 by replacing the standard convolution with ODConv in the shallow layers, thereby enhancing the model’s ability to extract small target features while also reducing the parameter count and computational cost to some extent. In the Neck, the addition of a dedicated detection head for small targets offers more possibilities for fusion paths. Moreover, the fusion strategy was improved by introducing a novel adaptive weighted fusion method. For the small target paths in the fusion process, a CBH structure was specifically designed, and the convolutional block attention module (CBAM)^34^ was incorporated to mitigate background interference. In the detection head, the ASFF technique was employed to further strengthen information fusion, leading to improved detection performance.

### Backbone network improvements

In the detection head section of the network, a four-head structure is adopted, and ASFF is used to replace the original YOLO detection head. The application of ASFF further promotes the fusion of multi-scale information, significantly enhancing detection outcomes. These improvements allow EFCNet to exhibit exceptional performance in processing complex images, especially in the detection of small objects.

**Fig. 3:**
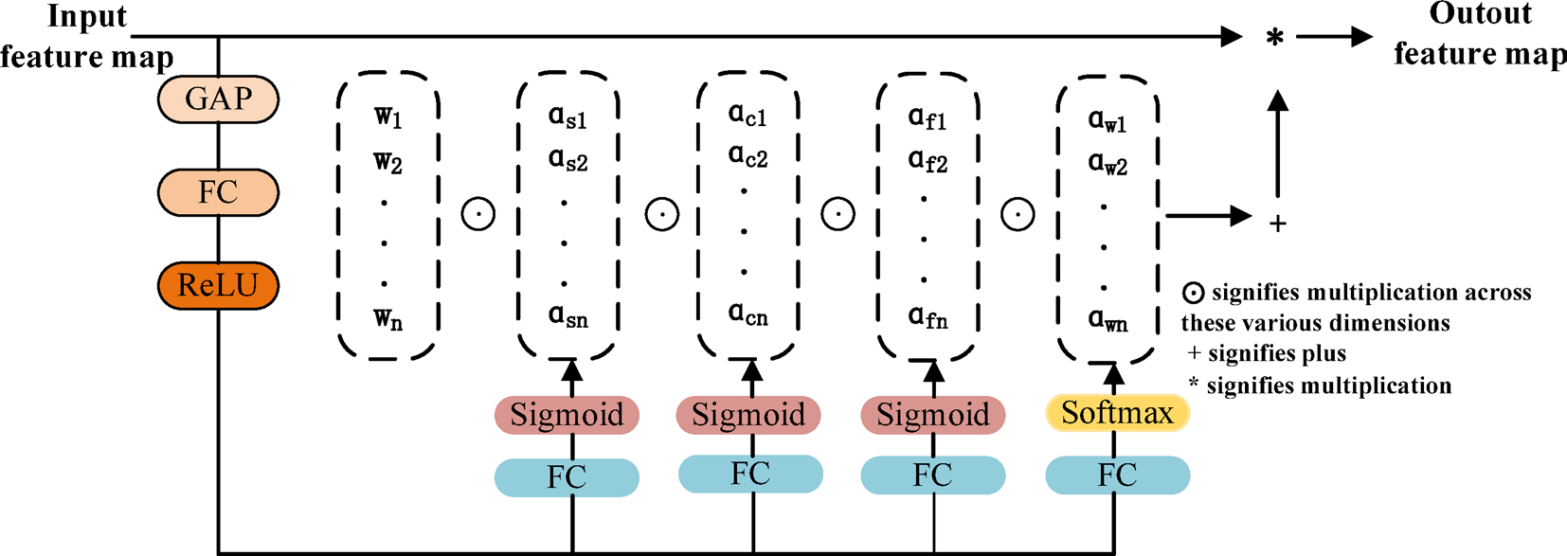
Omni-dimensional dynamic convolution structure.

The ODconv structure is shown in Figure.3, the input feature map is initially processed through global average pooling (GAP) to transform it into a 1 × 1×Cin dimension, thereby reducing dimensionality and computational complexity, in theprevious text, " Cin " refers to the output in terms of the channel dimension. After being compressed through a fully connected (FC) layer, the features are subjected to a non-linear transformation via the ReLU activation function to eliminate negative values. This structure is further divided into four branches: the first branch predicts the spatial position weights of each convolutional kernel, aiding in object localization; the second branch predicts the weights of the input channels, analysing the structural features of objects within these channels; the third branch measures the weights of the output channels to ensure the integrity of feature information during the convolution process; the fourth branch predicts the weights of the convolutional kernels, refining the capture of local features specific to different object categories. These mechanisms optimize the network’s ability to extract detailed information and enhance its detection capabilities for small objects. The output formula for ODConv is as follows:

1 $${\text{y}}=(\sum\limits_{{i=1}}^{n} {{\alpha _{si}} \odot {\alpha _{ci}} \odot {\alpha _{fi}} \odot {\alpha _{wi}} \odot {w_i}} ) \times x$$

In the formula, x represents the input features, and y represents the output features. The terms $\:{a}_{wi}$, $\:{a}_{si}$, $\:{a}_{ci}$, and $\:{a}_{fi}$ represent the attention scalars for the convolutional kernel $\:{W}_{i}$ along the spatial dimension, input channel dimension, and output channel dimension, respectively. The symbol ⊙ signifies multiplication across these various dimensions.

### Neck improvements

Given the limited integration and extraction of detailed and semantic information by the original three-layer PAN-FPN in YOLOv5, adding an additional minuscule object detection layer to extract more feature information, along with the introduction of a bidirectional feature pyramid network (BiFPN), can enhance the fusion of features at various scales. Although using BiFPN fusion nodes to integrate shallow and deep features results in feature maps rich in both detail and semantic information, the integration of deep features with shallow ones can inevitably introduce background details that may interfere with object detection. This problem is particularly pronounced in the processing of remote sensing images with significant detection interference, especially where the object blends with similarly textured backgrounds or where the background blurs the object, potentially diminishing the advantages of BiFPN and four-layer detection and consequently reducing the model’s detection performance. To address this, the paper redesigns the feature fusion nodes, proposing an enhanced small object detection oriented bidirectional feature pyramid.

**Fig. 4:**
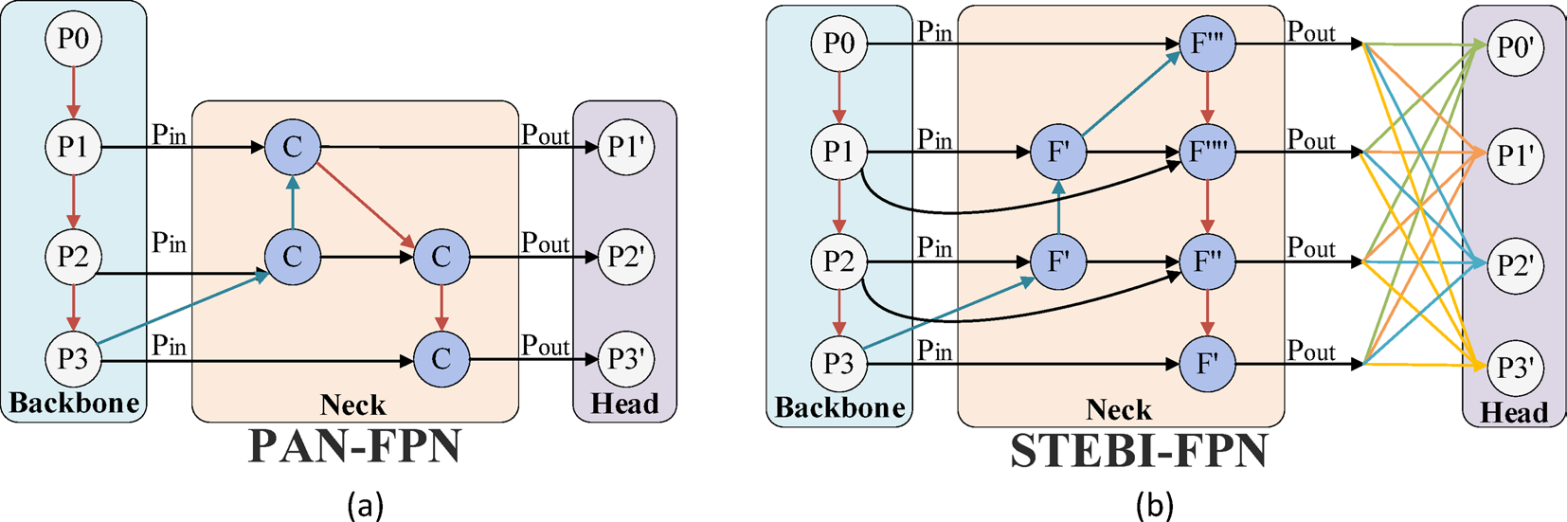
Figure (**a**) shows the structure of PAN-FPN, and figure (**b**) shows the structure of STEBI-FPN.

### Efficient multi-scale attention module

Figure. 4(a) and Figure. 4(b) illustrate the frameworks of PAN-FPN and STEBIFPN, respectively. In the diagrams, C represents the use of the original concat structure, while F represents the use of a completely new fusion approach, red lines represent 2x downsample fusion paths, blue lines represent 2x upsample fusion paths, and black lines indicate conventional fusion paths. The more downsampling a feature map undergoes, the richer the semantic information it contains but the sparser the detail information it retains. Therefore, after multiple downsampling processes, small objects carrying less spatial detail information may lose features representative of themselves.

Compared to PAN-FPN, STEBIFPN introduces an ultra-small object detection layer and spans two new feature fusion paths between the neck output end and the backbone output end. The ultra-small object detection layer not only incorporates the output feature map from the most information-rich and shallowest C3 module of the backbone network but also extends the feature extraction path of the neck network, allowing the last three detection layers to gather more semantic information. The two newly added fusion paths enable the neck network to acquire more detailed information.

**Fig. 5:**
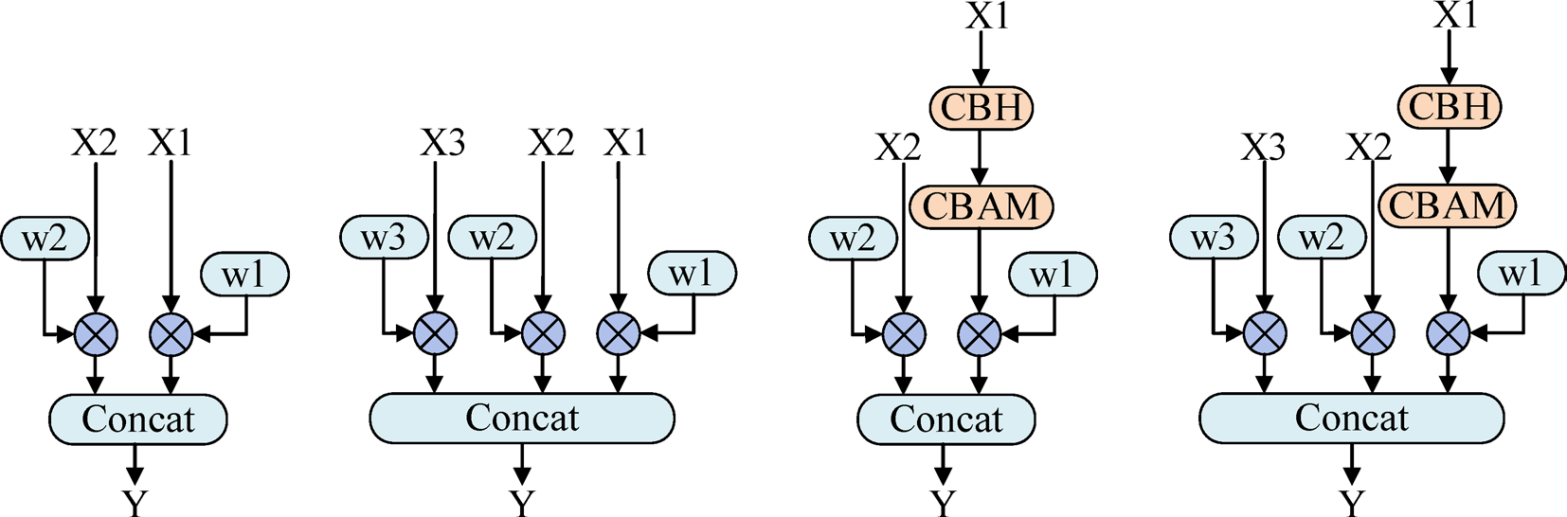
Diagram of the new fusion method structure.

Moreover, all fusion nodes in PAN-FPN employ a method of direct concatenation of feature map channels for fusion, while the fusion nodes in STEBIFPN are divided into four different structures, as shown in Figure. 5.

Figure. 5 displays fusion nodes with two and three input ports, where X1 to X3, serving as input feature maps to the fusion nodes, represent the output feature maps from shallow to deep layers. X1, containing the richest detail information, is processed through the CBH and CBAM attention modules to enhance object features and reduce background interference. Subsequently, X1 and other input feature maps are multiplied by the weighted coefficients $\:{W}_{i}$ to adjust the importance of the feature maps before being concatenated for fusion. The initial value of $\:{W}_{i}$ is set to 1, and it can be adaptively adjusted through training to accommodate learning.

**Fig. 6:**
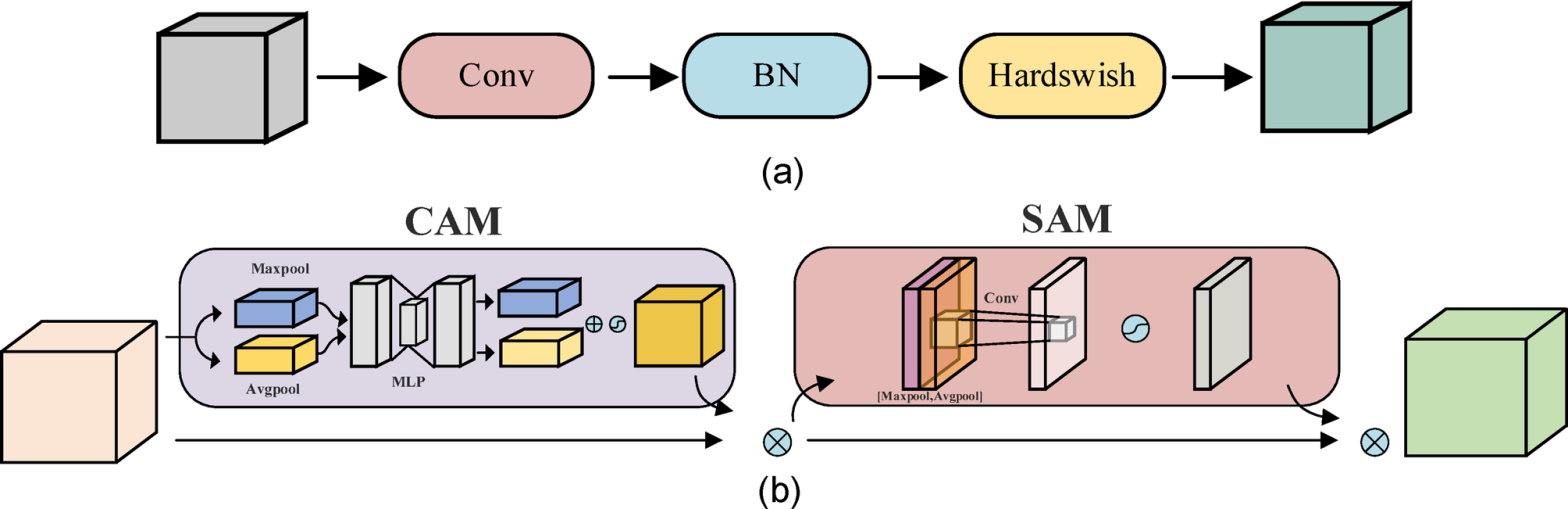
Figure (**a**) shows the structure of CBH and figure (**b**) shows the structure of CBAM.

Figure. 6(a) illustrates the CBH module, which is composed of a two-dimensional convolution layer, a batch normalization layer, and a Hardswish activation function. The CBH module significantly enhances the quality and expressive capability of the feature map by effectively extracting spatial features through convolution operations. The batch normalization layer standardizes the feature distribution, enhancing the model’s generalization ability. The Hardswish activation function introduces non-linearity, further boosting the network’s processing capabilities, enabling the overall network to more effectively learn and represent complex patterns in the data.

Figure. 6(b) displays the structure of the CBAM attention mechanism, which consists of a channel attention mechanism (CAM) and a spatial attention mechanism (SAM). Initially, the input feature map is processed through CAM to enhance the representation of objects at the channel level. Then, SAM enhances the spatial representation capability to reduce background interference. Specifically, CAM performs global max pooling and average pooling on the input feature map, followed by an MLP to compute two sets of weights. These weights are then added together and processed through a sigmoid activation function to obtain a channel-wise weight vector for the input feature map. This weight vector is multiplied by the input feature map channel-wise to produce a new set of feature maps. SAM then performs further spatial dimension analysis on the feature maps processed by CAM. Specifically, SAM uses a channel-wise max pooling and average pooling strategy on the feature maps output by CAM, generating two independent spatial attention maps. These spatial attention maps are stacked channel-wise and input into a small convolution layer, which contains only one convolution kernel to integrate spatial information from different pooling operations. The feature maps processed by this convolution layer generate the final spatial attention map through a sigmoid activation function. This map explicitly indicates which spatial areas in the feature map are key, thereby aiding the network in focusing on processing information from these areas.

### Detection head improvements

**Fig. 7:**
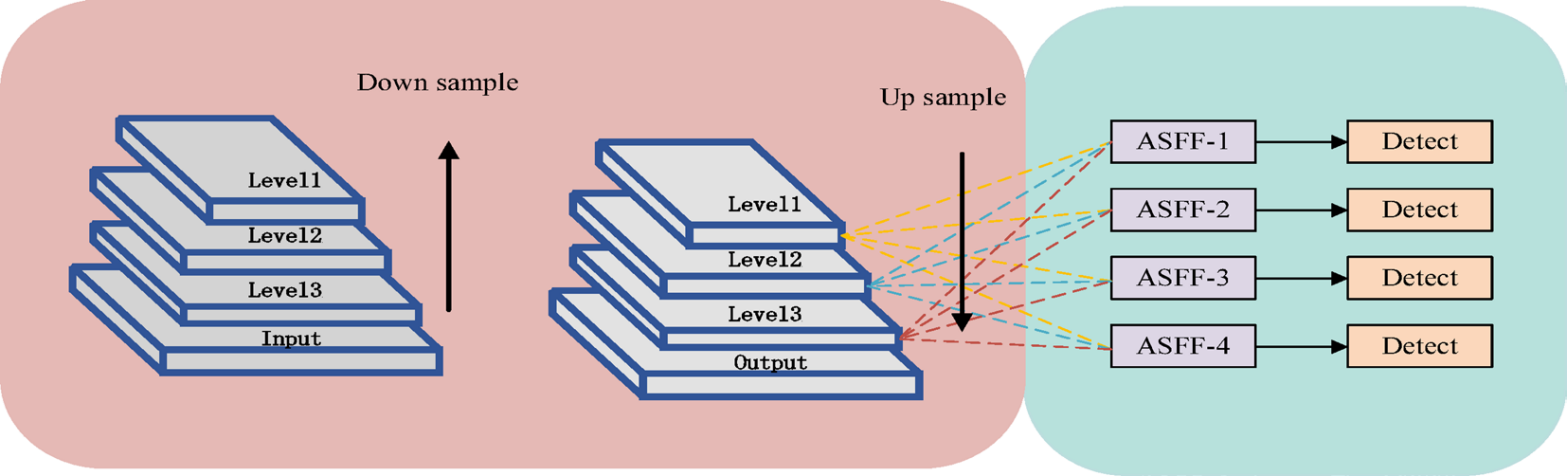
ASFF detection head structure diagram.

Due to the complexity of remote sensing images and the presence of information across various scales, a four-headed ASFF structure has been integrated into the model to enhance its generalization ability through multi-scale adaptive fusion. The structure is illustrated in the Figure. 7.

In the Figure. 7, Level1, Level2, Level3, and Level4 correspond to the feature maps output from the network’s Neck. The output of ASFF1 is obtained by adding the features from Level1 to Level4, each weighted appropriately. Similarly, the inputs and outputs for ASFF2, ASFF3, and ASFF4 are processed in the same manner as ASFF1. The computational formula is as follows:

2 $${\text{y}}_{{ij}}^{l}=\alpha _{{ij}}^{l} \times {\text{X}}_{{ij}}^{{1 \to l}}+\beta _{{ij}}^{l} \times {\text{X}}_{{ij}}^{{2 \to l}}+\gamma _{{ij}}^{l} \times {\text{X}}_{{ij}}^{{3 \to l}}+\kappa _{{ij}}^{{4 \to l}} \times {\text{X}}_{{ij}}^{{4 \to l}}$$

In the formula, $\:{y}_{ij}^{l}$ represents the output of the ASFF network, while $\:{x}_{\text{i}j}^{1\to\:l}, {x}_{\text{i}j}^{2\to\:l}, {x}_{\text{i}j}^{3\to\:l}$,$\:{x}_{\text{i}j}^{4\to\:l}$denote the feature vectors input at the corresponding positions. The terms $\:{\alpha\:}_{\text{i}j}^{l}, {\beta\:}_{\text{i}j}^{l}, {\gamma\:}_{\text{i}j}^{l}, {\kappa\:}_{\text{i}j}^{l}$ are the learnable weights from Level1 to Level4 to layerl.

Since the output of the ASFF is in a summative form, the dimensions and the number of channels of the output features must be consistent. Therefore, the feature maps from Level 1 to Level 4 are processed through a 1 × 1 convolution to align the number of channels consistently and derive the weights α, β, γ, and κ. Finally, these weight parameters are normalized through a softmax layer to ensure that the sum of the four weight parameters equals 1. The formula for a_$\:{a}_{\text{i}j}^{1}$ is as follows:

3 $$\alpha _{{ij}}^{1}=\frac{{{e^{\lambda _{{{\alpha _{ij}}}}^{l}}}}}{{{e^{\lambda _{{{\alpha _{ij}}}}^{l}}}+{e^{\lambda _{{{\beta _{ij}}}}^{l}}}+{e^{\lambda _{{{\gamma _{ij}}}}^{l}}}+{e^{\lambda _{{{\kappa _{ij}}}}^{l}}}}}$$

## Experiment results and analysis

### Dataset

**Fig. 8:**
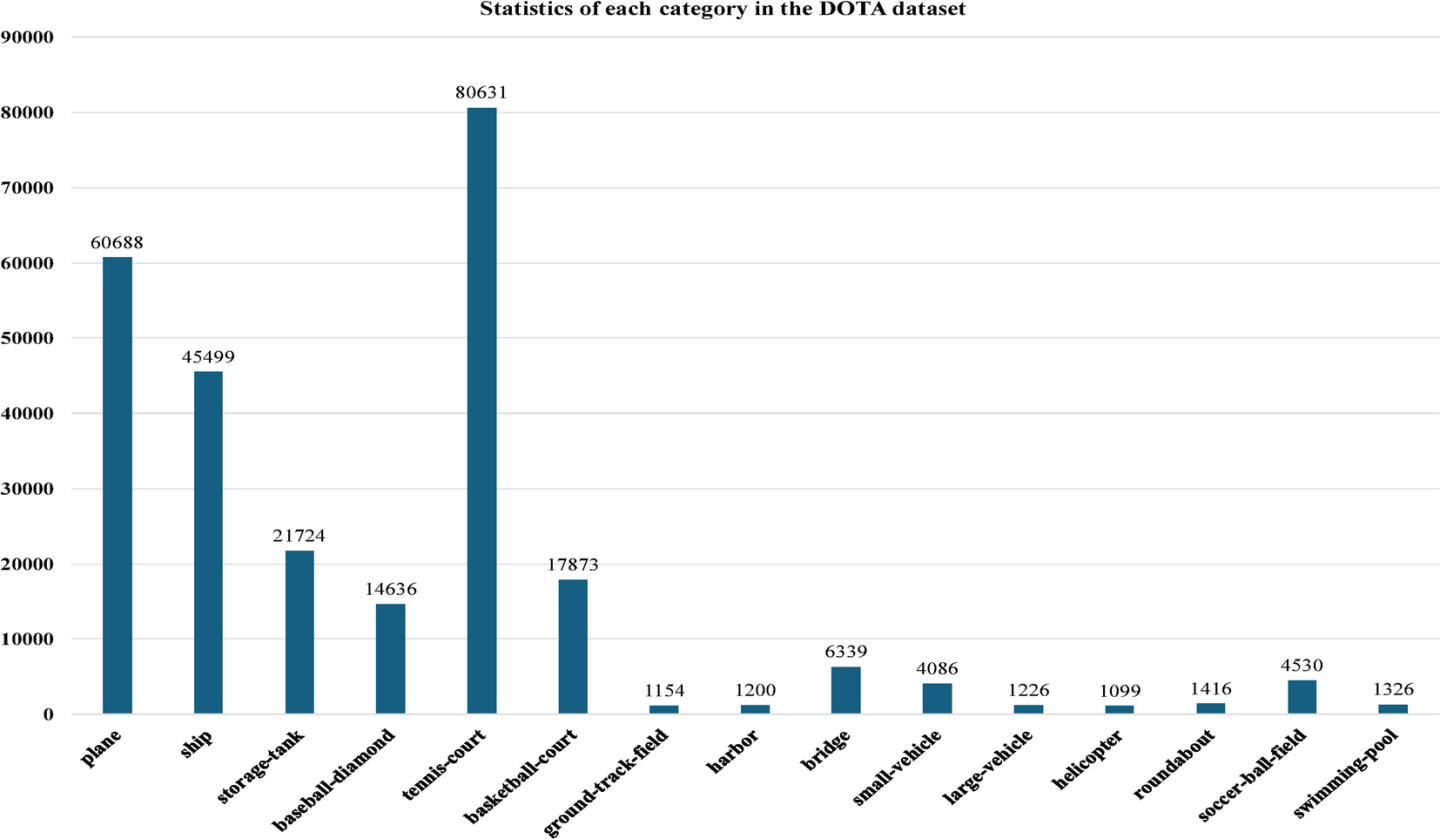
DOTA dataset target category statistics chart.

To assess the effectiveness of the improved model proposed in this paper, a series of experimental analyses were conducted using the challenging large-scale remote sensing object detection dataset DOTA V1.0. The DOTA V1.0 dataset comprises 2806 remote sensing images with resolutions ranging from 800 × 800 to 4000 × 4000, containing 188,282 object instances across 15 categories: small vehicles, large vehicles, airplanes, storage tanks, ships, harbors, ground runways, soccer fields, tennis courts, swimming pools, baseball fields, roundabouts, basketball courts, bridges, and helicopters. The total number of objects per category is illustrated in Figure. 8. To enhance the training effectiveness of the model, the original images were pre-processed through image segmentation and padding, expanding the dataset from 2806 images of varying resolutions to 21,046 images, with 15,749 used for training and 5,297 for testing.

In addition, to further validate the effectiveness of the proposed improvements, the DIOR dataset was used for evaluation. The DIOR dataset is characterized by its large-scale number of images and instances, as well as the diversity of object categories it covers. These categories include airplane, airport, baseball field, basketball court, bridge, chimney, dam, expressway service area, expressway toll station, harbor, golf course, ground track field, overpass, ship, stadium, storage tank, tennis court, train station, vehicle, and windmill.

### Experimental environment and training setup

The primary experimental setup for this study consists of an Ubuntu 20.04 operating system, PyTorch 2.0.0, Python 3.8, CUDA 11.8, and a 24GB RTX 4090 GPU. During the model training process, the resolution of all images was standardized to 640 × 640. The training utilized the stochastic gradient descent (SGD) optimizer along with a cosine annealing algorithm for weight adjustment. The Mosaic data augmentation method was used to enrich the dataset. The model’s learning rate was set at 0.01, the batch size for training images was 32, the number of training epochs was set at 300, and the complete intersection over union (CIOU) was selected as the loss function.

### Experimental evaluation indexes

In object detection, three types of metrics are commonly employed to evaluate model performance: Precision (P), Recall (R), and Mean Average Precision (mAP) serve as indicators of the model’s detection capabilities; Inference Time (IT) is used to assess the model’s detection speed; and GFLOPs and Parameters (Params) are used to evaluate the model’s deployment capabilities, where lower GFLOPs and Params suggest less demand on the hardware platform for deployment. Precision, Recall, and mAP can be calculated using the following formulas:

4 $$P=\frac{{TP}}{{TP+FP}}$$

5 $$R=\frac{{TP}}{{TP+FN}}$$

6 $$AP=\int\limits_{0}^{1} {P(R)dR}$$

7 $$mAP{\text{=}}\frac{{\sum\nolimits_{{i=1}}^{k} {A{P_i}} }}{k}$$

### Results and analysis of ablation experiment

To explore the optimal performance of the model on the DOTA dataset, we conducted an ablation study focusing on the IOU-thres and Conf-thres. The experimental results are shown in Table 1. After training YOLOv5 through all epochs, the model automatically performs validation, with the default IOU-thres and Conf-thres set to 0.6 and 0.001, respectively. Under these settings, the model achieves a mAP of 72.1%. Upon further tuning of the IOU-thres and Conf-thres, we found that when the IOU-thres was reduced to 0.4 while maintaining the Conf-thres at 0.001, both precision and recall reached their highest values, and the mAP also achieved its peak performance. This result suggests that the proper adjustment of IOU-thres and Conf-thres helps strike an effective balance between precision and recall, thereby improving the overall detection performance of the model.

**Table 1:** Ablation experiment schemes.

Conf-thres	IOU-thres	*P*	*R*	mAP
0.001	0.6	78.1%	66.9%	72.1%
0.25	0.5	78.2%	67.6%	74.9%
0.25	0.4	78.2%	67.9%	74.9%
0.2	0.5	78.2%	67.6%	75.0%
0.2	0.45	78.1%	67.9%	75.1%
0.2	0.4	78.1%	67.9%	75.1%
0.15	0.4	79.9%	67.9%	75.9%
0.1	0.4	78.2%	67.9%	75.2%

To verify the effectiveness of the improved model, ablation experiments were conducted based on the original YOLOv5s framework. The results of these experiments are shown in Table 2.

In Experiment A, the original YOLOv5s model was utilized for detection, with parameter size at 7.1 M, computational load at 15.9 GFLOPs, and inference time at 2.1ms. Compared to other models, it featured lower computational costs, but it also recorded the lowest mAP at 72.7%.

Experiment B implemented the ODCSP-Darknet53 as the backbone network, showing a slight decrease in computational load to 14.0 GFLOPs and an mAP increase of 0.8% over the original YOLOv5s, with an inference time of 2.8ms. This indicates that ODConv is more efficient at extracting shallow level information than standard convolution.

In Experiment C, the detection head was modified to use ASFF, resulting in an mAP increase to 74.2%, a significant improvement over YOLOv5s. However, the parameter size and computational load also increased to 12.5 M and 24.3 GFLOPs, respectively, with the inference time rising to 2.6ms. The results demonstrate a significant enhancement in object detection capabilities after modifying the detection head.

Experiment D introduced the STEBIFPN structure, achieving an mAP of 73.4%, a 0.7% increase over YOLOv5s, with parameters at 7.3 M and computational load at 19.6 GFLOPs. The inference time was 3.7ms. The results indicate that optimizing the fusion path and adopting an improved fusion method enhanced the model’s generalization and object detection capabilities.

Experiment E involved improvements to both the backbone network and the detection head, increasing the mAP to 75.0%, a 2.3% improvement over YOLOv5s. The parameter and computational loads were 12.5 M and 22.4 GFLOPs, respectively, with an inference time of 2.6ms. Compared to Experiment C, further enhancements in both modules resulted in improved information extraction capabilities and superior detection performance.

In Experiment F, ASFF was used as the detection head and the STEBIFPN module was added. The model achieved an accuracy of 75.4%, with a parameter count of 13.4 M. It had the highest computational cost among all models, reaching 32.1 GFLOPs, and an inference time of 3.5 ms. Compared to Experiments C and D, it showed a significant improvement in accuracy.

Experiment G showcased the model proposed in this paper, with parameters at 13.4 M, computational load at 30.2 GFLOPs, and an mAP of 75.9%, with an inference time of 3.8ms. Following optimizations to the backbone network, Neck fusion modules, and detection head, the model demonstrated substantial improvements over YOLOv5s. The interaction between various modules also enhanced the model’s capability for information extraction and feature map representation.

Table 3 Displays the detection results of various models on the DOTA dataset, with the best results in each category highlighted in bold. Analysis of the data reveals that the model improved in this study achieved the highest mAP value among all the refined models, reaching 75.9%. Particularly in the Bridge category, which is known for its detection difficulty, it significantly improved to 55.6% compared to other models. Additionally, this model achieved the highest mAP values in the detection of plane, bridge, Large-vehicle, ship, Tennis-court, harbor, and helicopter categories. Although the detection accuracy of this model did not reach the optimum in some categories, the differences in mAP values were not significant. Furthermore, the frames per second (FPS) of this model was also relatively high at 39, ranking among the higher levels compared to other models. Overall, the model proposed in this paper has im-proved both detection accuracy and speed, proving its applicability and effectiveness in detecting various small-sized object categories.

**Table 2:** Ablation experiment schemes on DOTA dataset.

Model	ODCSP-Darknet53	ASFF	STEBIFPN	mAP	Parameters(M)	GFLOPs	IT(ms)
A				72.7%	7.1	15.9	2.1
B	√			73.5%	7.1	14.0	2.8
C		√		74.2%	12.5	24.3	2.6
D			√	73.4%	7.3	19.6	3.7
E	√	√		75.0%	12.5	22.4	2.6
F		√	√	75.4%	13.4	32.1	3.5
G	√	√	√	75.9%	13.4	30.2	3.8

### Comparative tests on different datasets

**Table 3:** Comparison experiments on the DOTA dataset.

Category	RetinaNet-O ^35^	Faster-RCNN-O^36^	ARSD^37^	R2YOLOX-s ^38^	RIL ^39^	HOFA-Net^40^	CFC-Net^41^	FAMHE-Net^42^	RCSANet^43^	OURS
PL	88.67	88.44	86.90	88.2	88.94	90.42	89.08	89.62	89.89	**93.2**
BD	77.62	73.06	69.64	81.7	78.45	76.63	80.41	**83.74**	77.45	77.9
BR	41.81	44.86	46.38	42.3	46.87	47.57	52.41	53.84	54.61	**55.6**
GTF	58.17	59.09	56.85	61.7	72.63	59.01	70.02	**73.47**	65.11	65.7
SV	74.58	73.25	**80.60**	80.3	77.63	73.41	76.28	78.85	66.05	70.7
LV	71.64	71.49	66.96	78.7	80.68	85.64	78.11	83.42	80.12	**88.3**
SH	79.11	77.11	78.96	88.2	88.18	89.29	87.21	88.19	87.00	**90.0**
TC	90.29	90.84	90.76	90.9	90.55	90.76	90.89	90.91	90.88	**94.1**
BC	82.18	78.94	72.15	80.7	81.33	73.30	84.47	**87.55**	82.87	70.9
ST	74.32	83.90	78.96	86.2	83.61	**89.44**	85.64	85.75	86.92	81.0
SBF	54.75	48.59	39.67	48.2	64.85	**71.15**	60.51	63.59	60.89	55.8
RA	60.60	62.95	61.27	58.6	63.72	**69.39**	61.52	67.23	66.85	69.0
HA	62.57	62.18	72.23	73.5	73.09	75.16	67.82	75.65	77.62	**85.9**
SP	69.67	64.91	72.39	72.5	73.13	67.05	68.02	70.84	**75.20**	64.0
HC	60.64	56.18	50.64	52.0	56.87	74.77	50.09	58.6	71.5.	**76.2**
FPS	-	-	46	-	13.36	44.14	17.81	-	-	37.46
mAP	68.43	69.05	68.28	72.25	74.7	75.53	73.5	**76.7**	74.97	75.9

**Table 4:** Comparison experiments on the DIOR dataset.

	Parameters(M)	GFLOPs	mAP
YOLOv5s	7.0	15.8	74.5%
YOLOv7-tiny	6.0	13.1	74.7%
YOLOv7	36.6	103.5	80.2%
YOLOv8	3.0	8.0	78.4%
S^3^MKM^44^	13.1	41.6	74.3%
SuperYOLO^45^	7.7	20.9	71.8%
OURS	13.4	30.2	80.5%

In addition, to demonstrate the effectiveness of the improved model, we conducted comparative experiments on the DIOR^39^ dataset, as shown in Table 4. The comparison includes models from the YOLO series, such as YOLOv7 and YOLOv8, as well as other recent improved models. The results in the table show that our model achieved the highest overall accuracy of 80.5%, which is 0.3% higher than YOLOv7. At the same time, our model has significantly fewer parameters and lower computational complexity than YOLOv7. Although our model has slightly more parameters and computational complexity compared to other models, its overall mAP performance is superior.

### Visualisation of test results

**Fig. 9:**
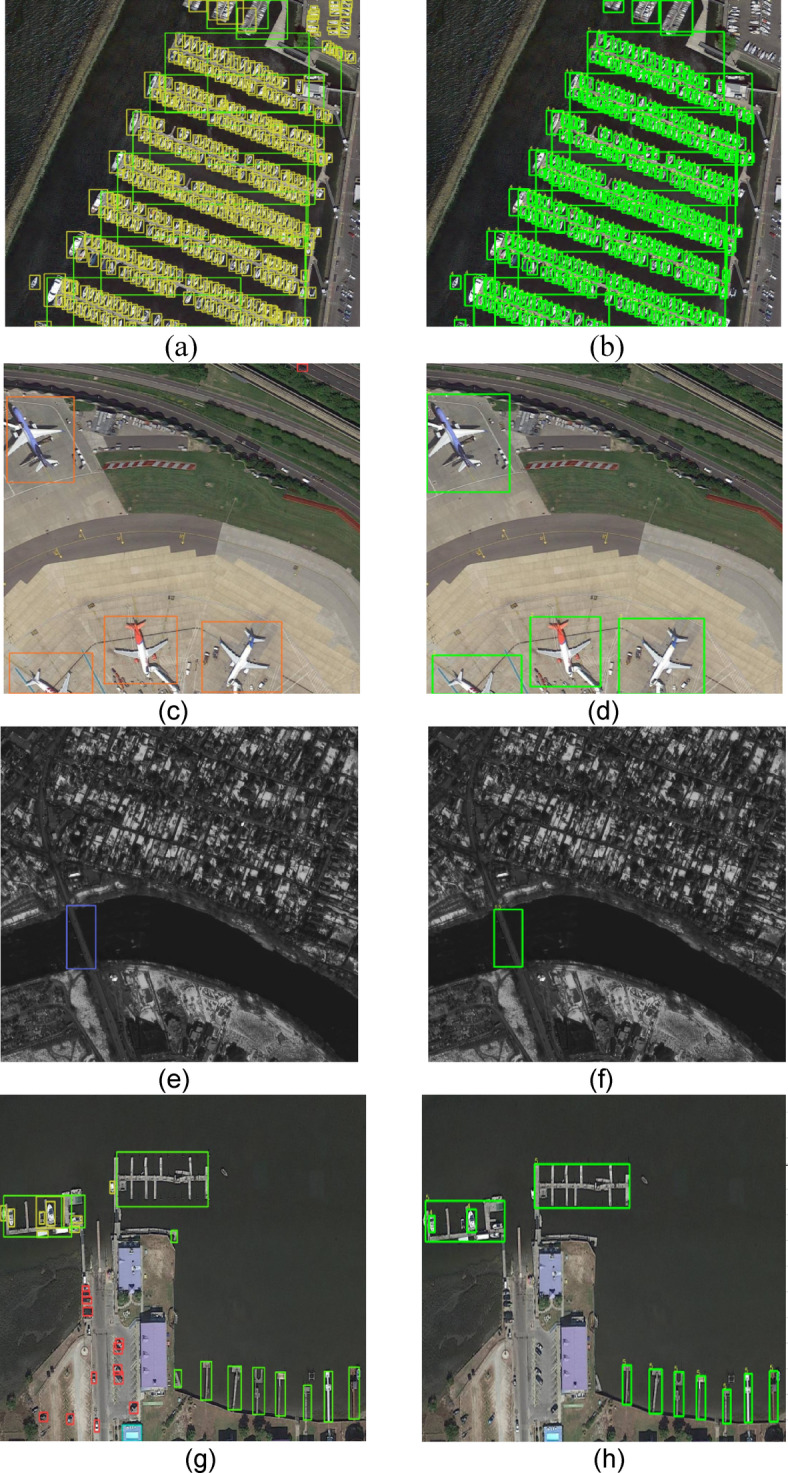
Visual detection graph of DOTA dataset, the left (**a**)-(**g**) are the visual detection results of EFCNet, and the right (**b**)-(**h**) are the actual annotation boxes.

To validate the improvements proposed in this study, we selected four distinct scenarios to demonstrate the detection performance of our model, as shown in Fig. 9. Figures 9(a), 9(c), 9(e), and 9(g) display the detection results produced by our model, while Figs. 9(b), 9(d), 9(f), and 9(h) present the actual annotation boxes. Figure 9(a) illustrates the model’s detection results for densely arranged small objects, where the model did not miss or misdetect any objects compared to the actual annotations. Notably, the model successfully identified an unannotated ship located in the upper right corner of the image. Figure 9(c) describes a scene from the DOTA dataset after object segmentation, where some object information was lost. Despite the airplane being truncated in the lower left corner, the model was still able to detect it accurately. Figure 9(e) demonstrates the detection of a bridge under low-light conditions, a particularly challenging task in the DOTA dataset, especially under adverse weather conditions. Nevertheless, the model accurately recognized the bridge within the image, showcasing the effectiveness of the improvements. Figure 9(g) illustrates the detection of various types of bridges against a more complex background, where the model did not miss any detections and was capable of identifying vehicles without external annotations, further validating its robustness.

**Fig. 10:**
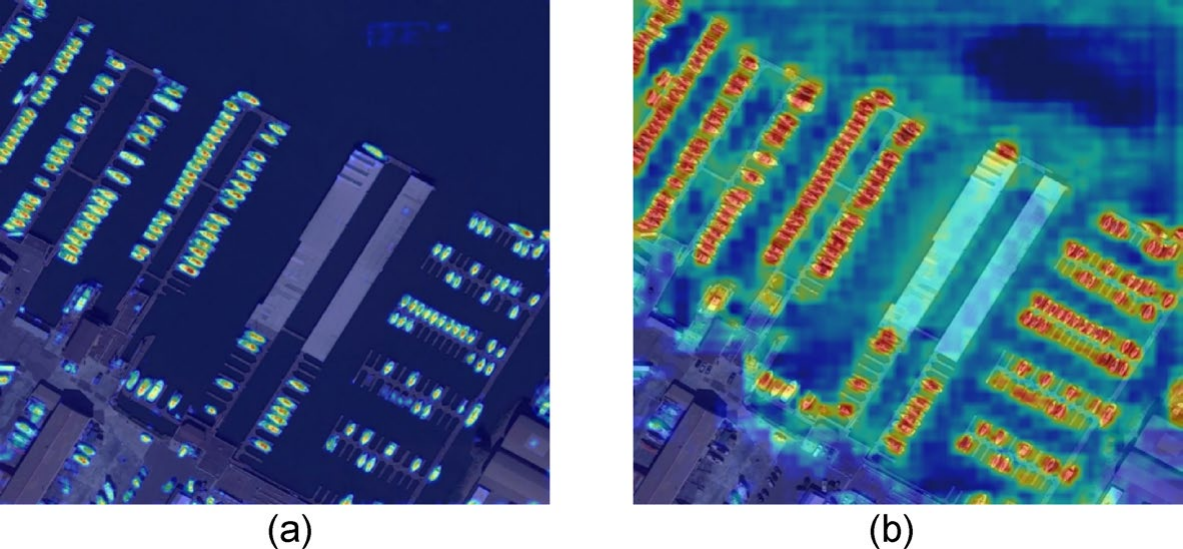
Comparison of heat maps between EFCNet and YOLOv5 in the DOTA dataset, The left figure (**a**) is the visual thermodynamic diagram of EFCNet, and the right figure (**b**) is the yolov5 visual thermodynamic diagram.

To further demonstrate the differences between the improved model developed in this study and the original YOLOv5s model, we utilized heatmaps to visualize the differences in model attention between the two. As shown in Figure. 10(a), the heatmap of the improved model from this study is displayed, while Figure. 10(b) shows the heatmap results of the original YOLOv5s model. Comparing these two images, it is evident that the attention of the model proposed in this study is more focused on the detection objects. In contrast, the attention of the original YOLOv5s model is more dispersed, particularly within the red-boxed area in Figure. 10(b), where the original model failed to effectively focus on the object.

These results indicate that the modifications introduced in the backbone network and the CBAM attention mechanism in the neck of the improved model effectively reduce the impact of background noise on the model and enhance its focus on object in-formation, which helps improve detection performance. The implementation of these improvement strategies not only optimizes the model’s focus but also enhances its ability to recognize objects in complex scenarios.

**Fig. 11:**
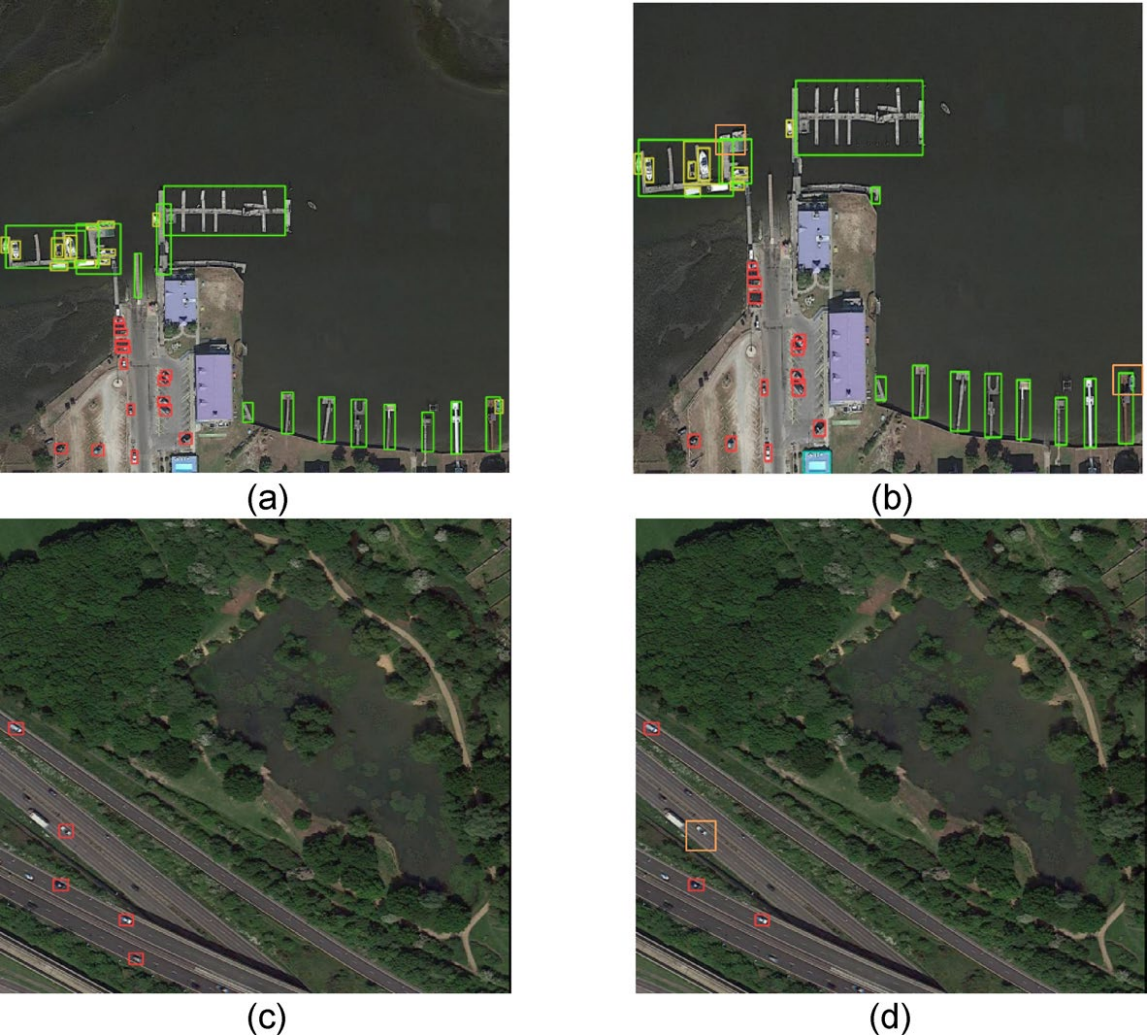
Comparison of DOTA dataset in different scenarios.

Furthermore, to more intuitively demonstrate the differences between EFCNet and YOLOv5, two sets of comparison images are presented in Fig. 11. The left side shows the detection results of the proposed model, while the right side displays those of YOLOv5. In the comparisons between subfigures (a) and (b), YOLOv5 exhibits missed detections, as indicated by the orange bounding box in subfigure (b). Similarly, in subfigures (c) and (d), YOLOv5 also fails to detect several vehicles. In contrast, EFCNet demonstrates superior detection capabilities, highlighting the effectiveness of the proposed improvements.

## Conclusions

This paper builds upon the YOLOv5s framework to enhance network capabilities for the detection of small objects in remote sensing imagery. The modified network structure uses ODCSP-Darknet53 as the backbone, which strengthens the model’s ability to extract detailed information from small objects. Additionally, to optimize the fusion of multi-scale information, a new fusion pathway and method have been adopted, and a detection head specifically designed for small objects has been added. These modifications achieve good detection results without significantly increasing the model’s parameter and computational demands. Moreover, the original detection head was replaced with an ASFF detection head to further enhance the model’s detection capabilities through multi-scale information fusion. Ablation studies on the DOTA dataset have demonstrated the effectiveness of each module, with the EFCNet outperforming other improved models, achieving an mAP of 75.9% with parameters and computational demands of 13.4 M and 30.2 GFLOPs, respectively, and a relatively high FPS. Thus, this provides an effective method for the detection of small objects in remote sensing imagery. However, our model still has limitations. Although the accuracy has been improved, the introduction of additional parallel branches may negatively impact detection efficiency when deployed on edge computing devices. In the future, we will focus on further optimizing the model to enhance the detection accuracy of small targets and reduce the false detection rate, with the goal of achieving better detection performance.

## Electronic supplementary material

Below is the link to the electronic supplementary material.

Supplementary Material 1

## Data Availability

The data generated during and/or analyzed during the current study are available from the corresponding author on reasonable request.
