# Supplementary material for: EFCNet for small object detection in remote sensing images
Source: Sci Rep. 2025 Jul 1;15:20393. doi: 10.1038/s41598-025-09066-z (PMC12219830; doi:10.1038/s41598-025-09066-z)
Supplement: Supplementary file 1 — Supplementary Material 1 [file 41598_2025_9066_MOESM1_ESM.docx]

**Appendix**

| Full term | Abbreviation |
| --- | --- |
| convolutional neural network | CNN |
| partial hybrid dilated convolution | PHDC |
| regin attention | RA |
| sigmoid linear unit | SiLU |
| exponential linear unit | ELU |
| omni-dimensional dynamic convolution | ODConv |
| small object enhancement bi-directional feature pyramid network | STEBIFPN |
| adaptively spatial feature fusion | ASFF |
| enhanced feature convergence network | EFC-Net |
| convolutional block attention module | CBAM |
| global average pooling | GAP |
| fully connected | FC |
| bidirectional feature pyramid network | BiFPN |
| channel attention mechanism | CAM |
| spatial attention mechanism | SAM |
| stochastic gradient descent | SGD |
| complete intersection over union | CIOU |
| frames per second | FPS |
